# Human Infection with *Rickettsia* sp. related *to R. japonica*, Thailand

**DOI:** 10.3201/eid1304.060585

**Published:** 2007-04

**Authors:** Jariyanart Gaywee, Piyanate Sunyakumthorn, Wuttikon Rodkvamtook, Toon Ruang-areerate, Carl Jeffries Mason, Narongrid Sirisopana

**Affiliations:** * Armed Forces Research Institute of Medical Sciences, Bangkok, Thailand

**Keywords:** Rickettsiosis, *Rickettsia japonica*, spotted fever, tick-borne rickettsiae, febrile illness, indirect fluorescence assay, polymerase chain reaction, nucleotide sequence analysis, Thailand, letter

**To the Editor:** Although rickettsioses caused by scrub typhus and typhus group rickettsiae are well recognized in Thailand, few spotted-fever group rickettsiae (SFGR), including *Rickettsia honei* TT118 and *R. felis,* have been documented to be associated with human illnesses (*1*,*2*). We report a case of human infection with an SFGR species closely related to *R. japonica* in Thailand.

In January 2005, a 36-year-old man with prolonged fever, pneumonia, and septic shock was transferred from a private hospital to Phramongkutklao Army Hospital in Bangkok. Two weeks before the onset of fever, the patient had camped at Khao Yai National Park, ≈175 km northeast of Bangkok. The park is a popular location for tourists and the largest national park declared as a natural wildlife reserve area. The patient reported the presence of wild deer around the camping area but did not recall being bitten by an arthropod. Ten days before hospitalization, he developed flulike symptoms, fever, and sore throat. Six days later, he noted petechiae on his lower extremities, and his condition worsened. At the time of hospital admission, the patient had fever of 38.6°C, tachycardia, dyspnea, hypotension, nausea, vomiting, generalized maculopapular rash, and subconjunctival hemorrhage. Laboratory investigation showed thrombocytopenia (platelets 64,000/mm^3^), leukocytosis (14,000/mm^3^), and elevated levels of serum hepatic enzymes (aspartate aminotransferase 287 IU/L [reference 5–50 IU/L]; alanine aminotransferase 186 IU/L [reference 5–40 IU/L]). Chest radiograph showed interstitial pneumonitis. Serum antibody test results were negative for leptospira and dengue virus; blood smear was negative for malaria.

Samples of the patient’s whole blood were collected in EDTA on days 10, 18, 20, and 25 after illness onset, and each sample was sent at the time of collection to the Armed Forces Research Institute of Medical Sciences, Bangkok, to be investigated for rickettsial infection. Plasma was separated and tested for scrub typhus, typhus group, and SFGR-specific immunoglobulin M (IgM) and IgG by immunofluorescence assay by using *Orientia tsutsugamushi* Karp-Kato-Gilliam strains and *R. typhi* Wilmington and *R. honei* TT118 whole cell antigens. No antibodies to rickettsiae were detected in the initial sample. On day 18, only antibodies against *R. honei* TT118 antigen were detected at a low titer, 50 for IgM and 200 for IgG, while antibodies to scrub typhus and typhus group rickettsiae remained negative (titers <50). Antibody level was unchanged on days 20 and 25.

At the time of admission, the patient began receiving 2 g of intravenous ceftriaxone and 200 mg of oral doxycycline daily. Three days later, treatment with doxycycline was stopped because the initial serologic results for rickettsia were negative. However, doxycycline was resumed on day 21, after antibodies to Thai tick typhus agent were detected in a second specimen. Within 3 days, the patient was afebrile and asymptomatic. He was discharged from the hospital and continued oral doxycycline for an additional 7 days. At 2-week follow up, he had completely recovered.

To identify which SFGR was responsible for the patient's illness, we used molecular approaches. We extracted DNA from the patient’s blood specimens by using QIAamp Mini blood kit (QIAGEN, Valencia, CA, USA) and subjected it to duplex nested–PCR assays targeting a 343-bp fragment of the rickettsial genus–specific 17-kDa antigen gene (*3*) and a 690 bp-portion of the *Orientia* 56-kDa antigen gene (*4*). An appropriate control panel included DNA from a reference sample of human blood, *Coxiella* sp., and *Leptospira interrogans*. Platinum *Taq* DNA Polymerase High Fidelity (Invitrogen, Carlsbad, CA, USA) enzyme mixture was used in PCR. By resolution on agarose gel, a PCR fragment of the expected size for the 17-kDa antigen gene was observed from the day-10 sample but not from the control samples. *Alu*I restriction pattern of amplified 17-kDa fragment was similar to that of SFGR. Additional rickettsial gene fragments, 630 nt-*ompA* (nt 70–701) and 945 nt-*gltA* (RpCS.193F-5′-GTAGGGTATCTGCGGAAGCC-3′, RpCS.1143R-5′-GAGCGAGAGCTTCAAGTTCTATTGC-3′), were also amplified from the day-10 specimen. All amplicons were excised from agarose gels, purified by QIAEX II Gel Extraction Kit (QIAGEN), and then sequenced. BLAST analysis of 17-kDa antigen gene (GenBank accession no. DQ909071), *gltA* (DQ909073), and *ompA* (DQ909072) segments obtained from this patient showed 99% identity to corresponding genes of *R. japonica*. Phylogenetic analysis of these 3 genes indicates that the *Rickettsia* sp. from this patient is closely related and clustered within the same clade of *R. japonica* (Figure). Isolation of this rickettsial agent from the patient’s blood by animal inoculation and by cell culture methods is ongoing.

**Figure:**
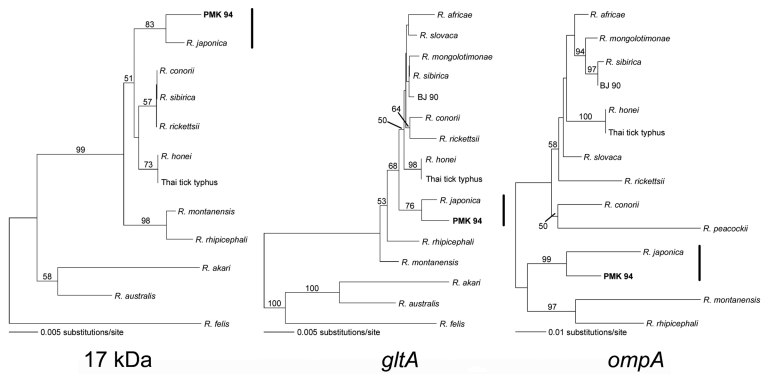
Phylogenetic relationships between *Rickettsia* sp. and rickettsial genes amplified from the patient (PMK 94) inferred from comparison with the rickettsial 17-kDa antigen gene, *gltA*, and *ompA* sequences by the neighbor-joining method. Bootstrap values of 1,000 replicates are indicated.

Persons visiting Khao Yai National Park are at risk for rickettsioses, particularly SFGR. Vectors for SFGR have been found in this area (*5*). The clinical and molecular findings in this case add to the accumulating data on the emerging rickettsial agents and their geographic distribution in Thailand.
